# IDH-mutant gliomas in children and adolescents - from biology to clinical trials

**DOI:** 10.3389/fonc.2024.1515538

**Published:** 2025-01-06

**Authors:** Louise Evans, Sarah Trinder, Andrew Dodgshun, David D. Eisenstat, James R. Whittle, Jordan R. Hansford, Santosh Valvi

**Affiliations:** ^1^ Michael Rice Centre for Hematology and Oncology, Women’s and Children’s Hospital, North Adelaide, SA, Australia; ^2^ Kids Cancer Centre, Sydney Children’s Hospital, Sydney, NSW, Australia; ^3^ Children’s Cancer Institute, Lowy Cancer Research Centre, University of New South Wales Sydney, Sydney, NSW, Australia; ^4^ Department of Pediatrics, University of Otago, Christchurch, New Zealand; ^5^ Children’s Hematology/Oncology Centre, Christchurch Hospital, Christchurch, New Zealand; ^6^ Children’s Cancer Centre, Royal Children’s Hospital, Melbourne, VIC, Australia; ^7^ Department of Stem Cell Medicine, Murdoch Children’s Research Institute, Melbourne, VIC, Australia; ^8^ Department of Pediatrics, University of Melbourne, Melbourne, VIC, Australia; ^9^ Department of Medical Oncology, Peter MacCallum Cancer Centre, Melbourne, VIC, Australia; ^10^ Personalized Oncology Division, Walter and Eliza Hall Institute (WEHI), Parkville, VIC, Australia; ^11^ Department of Medical Biology, University of Melbourne, Parkville, VIC, Australia; ^12^ Pediatric Neuro-Oncology, Precision Cancer Medicine, South Australia Health and Medical Reseach Institute, Adelaide, SA, Australia; ^13^ South Australia ImmunoGENomics Cancer Institute, University of Adelaide, Adelaide, SA, Australia; ^14^ Department of Pediatric and Adolescent Oncology/Hematology, Perth Children’s Hospital, Nedlands, WA, Australia; ^15^ Brain Tumor Research Program, Telethon Kids Institute, Nedlands, WA, Australia; ^16^ School of Medicine, Division of Pediatrics, The University of Western Australia, Perth, WA, Australia

**Keywords:** IDH mutation, low grade glioma, pediatric, adolescent and young adult, AYA

## Abstract

Gliomas account for nearly 30% of all primary central nervous system (CNS) tumors in children and adolescents and young adults (AYA), contributing to significant morbidity and mortality. The updated molecular classification of gliomas defines molecularly diverse subtypes with a spectrum of tumors associated with age-distinct incidence. In adults, gliomas are characterized by the presence or absence of mutations in isocitrate dehydrogenase (*IDH*), with mutated *IDH* (mIDH) gliomas providing favorable outcomes and avenues for targeted therapy with the emergence of mIDH inhibitors. Despite their rarity, *IDH* mutations have been reported in 5-15% of pediatric glioma cases. Those with primary mismatch-repair deficient mIDH astrocytomas (PMMRDIA) have a particularly poor prognosis. Here, we describe the biology of mIDH gliomas and review the literature regarding the emergence of mIDH inhibitors, including clinical trials in adults. Given the paucity of clinical trial data from pediatric patients with mIDH glioma, we propose guidelines for the inclusion of pediatric and AYA patients with gliomas onto prospective trials and expanded access programs as well as the potential of combined mIDH inhibition and immunotherapy in the treatment of patients with PMMRDIA at high risk of progression.

## 1 Introduction

Gliomas are a complex and diverse group of central nervous system (CNS) neoplasms that arise from glial cells, varying in location, grade, and clinical behavior (1–3). These tumors are particularly common in pediatric and adolescent and young adult (AYA) patients, comprising 30% of all CNS tumors and significantly contributing to morbidity and mortality (2, 4–7). The evolution of the integrated histo-molecular classification of gliomas defines tumors according to molecular features that influence patient prognosis and therapeutic decisions. In adults, the identification of isocitrate dehydrogenase (*IDH*) mutations has resulted in paradigm shift in the classification of tumors, with the recognition that these features play an important role in tumorigenesis and prognosis (7); however, less is understood on the role of mIDH in pediatric and AYA patients.

IDH enzymes are essential in major cellular metabolic processes (8, 9), and exist in three isoforms: IDH1, IDH2, and IDH3 (10, 11) with *IDH1* and *IDH2* mutations resulting in the accumulation of the oncometabolite D2-hydroxyglutarate (D2-HG) that is implicated in tumorigenesis through a variety of mechanisms. Mutant IDH1 or IHD2 enzyme activity interferes with normal cellular metabolic processes by depleting α-ketoglutarate (α-KG) from the Krebs cycle and disruptions in redox balance (11–14). In addition, D2-HG results in epigenetic modulation and impaired DNA repair, as well as angiogenesis through hydroxylation of hypoxia-inducible factor- α (HIF- α) (11–14).

*IDH* mutations have been identified in numerous cancers, including CNS tumors, solid tumors, and myeloid malignancies (15–18). In gliomas, these were first described in the context of “secondary glioblastoma (GBM),” before defining lower grade gliomas highlighting prognostic and treatment implications (19–23). The historical standard of care for adults with mIDH glioma involves maximal safe resection followed by radiation and sequential chemotherapy based on the risk of recurrence, although some patients may be suitable for active surveillance (3, 24, 25). Given the toxicity conferred by radiation and chemotherapy, approaches that can delay this intervention are required. Several m*IDH* inhibitors have been tested in the clinic with the pivotal INDIGO study (26) demonstrating significant improvement in progression free survival (PFS) and time to next treatment intervention in a select group of patients who have not received radiation or chemotherapy. Although patients older than 12 years were eligible for the study, no pediatric patients were enrolled on the treatment arm and thus findings in pediatric patients with m*IDH* glioma are lacking. Moreover, outstanding questions regarding the biology and prognostic features of these tumors in pediatric and AYA cohorts is yet to be fully elucidated with no consensus for their management (21).

## 2 The biology of the IDH enzyme

### 2.1 IDH enzyme

IDH enzymes are crucial in several cellular metabolic processes, including the Krebs cycle, glutamine metabolism, lipogenesis, and regulation of cellular redox status (8, 9). Three IDH isoforms, IDH1, IDH2 and IDH3, encoded by different genes with distinct cellular localization, contribute to the regulation of central metabolic circuits (10–12). IDH1 is located in the cytoplasm and peroxisomes, whereas IDH2 and IDH3 are localized to the mitochondria (10, 22).

**Figure 1:**
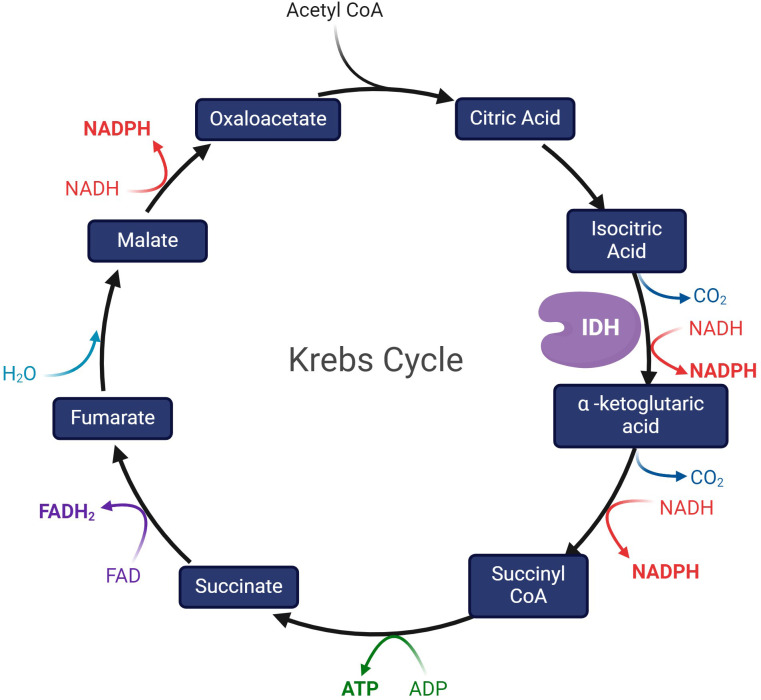
The Krebs cycle, depicting IDH as the enzyme that catalyzes the conversion of isocitric acid to α -ketoglutaric acid.

IDH enzymes are fundamental to the tricarboxylic acid cycle (TCA, Krebs cycle) (9): a series of metabolic oxidative reactions necessary for the mitochondrial electron transport chain to produce ATP (**Figure 1**) (6). Nicotinamide adenine dinucleotide phosphate (NADP)-dependent IDH1 and IDH2 catalyze the oxidative decarboxylation of isocitrate into α-ketoglutarate (α-KG), NAPDH, and carbon dioxide (CO2) (3, 9). α-Ketoglutarate is a major modulator of electron transport chain activity and TCA flux (27) and it is a co-factor of numerous important cellular reactions, including fatty acid metabolism (3, 10).

IDH is critical to maintaining a sufficient pool of reduced glutathione (GSH) and supporting the peroxiredoxin system by generating NADPH (12, 28). The NADPH produced from the reactions catalyzed by IDH1 and IDH2 is critical to maintain redox balance and protect cells from reactive oxygen species (ROS) that cause DNA damage (3, 10, 12, 29, 30). IDH is particularly important in the brain, producing 65% of the brain’s NADPH, which is essential for lipid metabolism (31). IDH1 and IDH2 are also involved in glutamine metabolism (32), which is important in tumorigenesis as glutamine deprivation suppresses cancer growth (28).

IDH3 is a holoenzyme located in the mitochondria and it catalyzes the irreversible nicotinamide adenine dinucleotide (NAD+)-dependent α-KG (6, 10, 11).

### 2.2 IDH mutations and tumorigenesis

**Figure 2:**
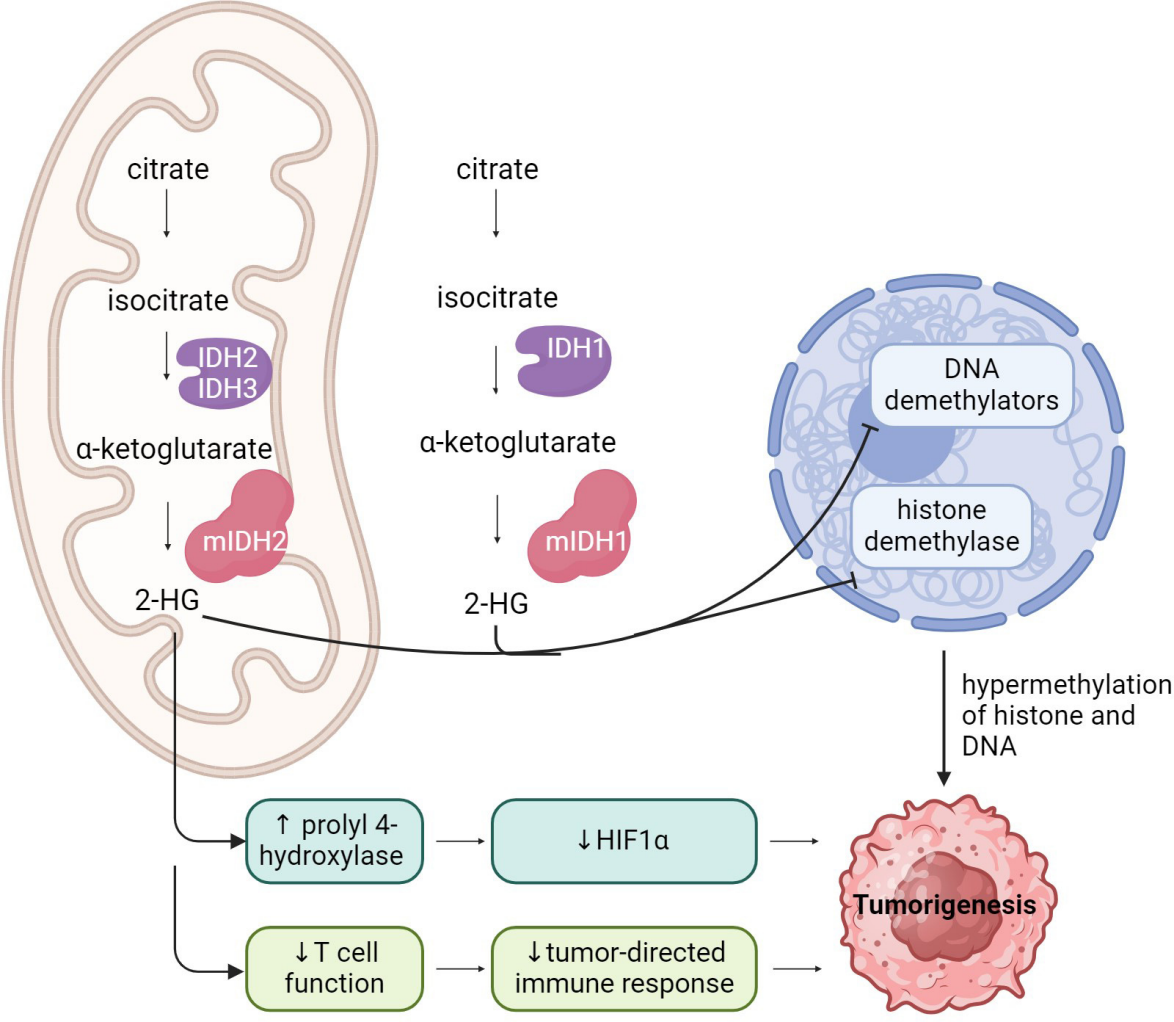
A schematic representation of IDH function, and the impact of IDH mutations on cell function, leading to tumorigenesis.

*IDH* mutations contribute to tumorigenesis in multiple cancer types by interfering with normal cellular metabolism and through the production of the oncometabolite D2-hydroxyglutarate (D2-HG) (**Figure 2**). Somatic mutations in *IDH1* and *IDH2* are heterozygous, and primarily consist of missense variants that result in single amino acid substitutions at key arginine residues within the enzyme’s core active sites (10, 11). *IDH1* mutations typically occur at Arginine 132, the R132H and R132C variants are the most prevalent (11). *IDH1* G97 is mutated in some colon cancers and pediatric astrocytomas (33). *IDH2* mutations occur at Arginine 172 and Arginine 140 (34, 35). There are no reports of tumor-associated mutations in the *IDH3* gene (10, 36).

Uncontrolled cell proliferation associated with cancer often leads to metabolic alterations that support rapid cell growth (37). Mutant IDH enzyme activity interferes with normal cellular metabolism by depleting α-ketoglutarate (α-KG) from the Krebs cycle (2). Mutant IDH enzymes also consume NADPH, reducing its availability for maintaining redox balance and *de novo* lipogenesis (38). Accumulating oxidative damage is a hallmark of cancer biology for *IDH*-mutated malignancies, as a direct consequence of disruption to redox balance (39). Under hypoxic conditions, *IDH1*-mutant cells exhibit increased oxidative TCA metabolism and decreased reductive glutamine metabolism (32, 40). Metabolically reduced glutamine serves as the major carbon source for fatty acid synthesis during hypoxia and impaired cellular respiration, a switch that is crucial for sustaining rapid cell proliferation (37).

In addition to losing its normal catalytic activity, the mutant IDH enzyme catalyzes the reduction of α-KG to its (R)-enantiomer, the oncometabolite D2-HG (9–13, 41–43). Accumulation of D2-HG causes profound metabolic dysregulation, including inhibition of normal cellular differentiation, epigenetic modulation, DNA repair, redox balance as well as alterations to the tumor immune microenvironment (11–14).

D2-HG is a homolog of α-KG, and functions as a competitive inhibitor of α-KG-dependent dioxygenases, vital for DNA and histone demethylation (3, 42, 44). Accumulation of D2-HG has been shown to inhibit several key histone demethylases, including KDM7A (demethylates H3K9me2 and H3K27me2), KDM4A/B (demethylates H3K9 and H3K36), and Jumonji C domain-containing (JmjC) histone demethylases (2, 11, 41, 44). D2-HG also inhibits the DNA modifying enzymes in the ten-eleven translocation (TET) family of 5-methlycytosine (5mC) hydroxylases (44). Interference with the normal activity of dioxygenases disrupts histone and DNA methylation patterns, leading to the signature global DNA hypermethylation phenotype seen in IDH-mutant gliomas, known as the Glioma CpG Island Methylator Phenotype (G-CIMP) (2). This epigenetic reprograming results in a block of cellular differentiation, which in turn causes inappropriate activation of growth-promoting signaling (2).

A high concentration of D2-HG also promotes angiogenesis via *VEGFR2* signaling and increased matrix metalloproteinase (*MMP2*) activity (45). Additionally, D2-HG contributes to gliomagenesis by directly stimulating prolyl 4-hydroxylase activity, thereby decreasing hypoxia-inducible factor- α (HIF1α) activity, which enhances the proliferation of human astrocytes *in vitro* (2, 46). Furthermore, tumor cell-derived R-2-HG is absorbed by T cells, leading to a disruption of nuclear factor of activated T cells (NFAT) transcriptional activity and polyamine biosynthesis, resulting in the suppression of T cell activity (47). D2-HG drives an immunosuppressive tumor microenvironment, acting directly on CD8+ cells by altering their metabolic and cytotoxic signatures (26, 47, 48).

D2-HG has been shown to accumulate in high levels in glioma cells but is absent in normal brain cells (44). D2-HG also structurally and functionally mimics glutamate, thereby contributing to the genesis of seizures in patients with gliomas (36, 49).

Patients with Ollier disease and Maffucci syndrome, non-hereditary skeletal disorders characterized by multiple enchondromas and spindle cell hemangiomas, are associated with somatic mosaic IDH1 and IDH2 mutations (50). These patients are at increased risk of developing IDH-mutant gliomas (51) and require thorough clinical examination along with a full body MRI every second year, from age 25 years (52).

IDH mutations have been implicated in other cancer types, including AML, myelodysplastic syndrome (15–18), cholangiocarcinoma of intrahepatic origin (53), central and periosteal cartilaginous chondrosarcomas (54), and melanoma (55, 56).

## 3 Pediatric gliomas

Traditionally, the World Health Organization (WHO) classification divides gliomas into four grades based on histology. Low grade gliomas (LGGs) are well-differentiated, typically slow-growing tumors (grades 1 and 2), whereas high grade gliomas (HGGs) are poorly differentiated or anaplastic, and diffusely infiltrating tumors (grade 3 and 4) (10). Recent advances in the understanding of molecular biology of CNS tumors were incorporated into the fifth edition of the WHO CNS tumor classification (CNS5), incorporating molecular characteristics into the diagnostic criteria in addition to the routine histologic features (57, 58).

Pediatric and AYA LGGs constitute most gliomas in this population, and tend to be more indolent, slow growing lesions. Treatment of LGG is multifaceted and is contingent upon several factors including tumor location, patient age and comorbidities. Whenever feasible, surgical resection is the preferred initial treatment. However, surgical resection is not always possible due to tumor location and surgical morbidity. When surgery is not an option, medical therapy choices include carboplatin as a single agent (59) or in combination with vincristine (60), or single agent vinblastine (61). Despite the intent to cure, treatment interventions can negatively impact neurological, neurocognitive and endocrine function, with long term effects on education, employment and social outcomes (4).

HGGs account for approximately 10% of brain tumors in children and adolescents and are more aggressive, conferring a poor prognosis (62). Standard treatment for HGGs include maximal safe resection, focal radiotherapy, and chemotherapy (62). Temozolomide forms an important part of treatment for adult HGGs concurrent to radiotherapy and as adjuvant therapy. However, in pediatric and AYA patients, the role of temozolomide is less clear given the tumor biology is different to that of adult HGGs.

### 3.1 IDH mutations in gliomas

*IDH1* and *IDH2* mutations are important, class-defining mutations in gliomas that carry significant therapeutic and prognostic implications (3, 63). *IDH* mutations are very early events in gliomagenesis, affecting a common glial precursor cell population in most cases except in patients with replication repair deficiency (RRD) (10, 64, 65), and show almost ubiquitous expression throughout the life-cycle of the disease. Among the three isoforms of IDH, mutations in *IDH1* are the most common (66).

The latest WHO Classification of CNS Tumors divides mIDH adult-type diffuse gliomas into astrocytoma grades 2-4 and oligodendroglioma 1p/19q-codeleted. Mutations in *IDH1* or *IDH2* define WHO grade 2 and 3 diffuse gliomas in adults (5, 34, 66, 67).

Patients with IDH-mutant gliomas tend to be younger than their wild-type counterparts with a median age of 37 years (68). *IDH1* gliomas arise from a neural precursor population that is spatially and temporally restricted in the brain (69). Therefore, mIDH tumors tend to be in the frontal lobes compared with other lobes and compared with *IDH* wild-type tumors (70).

The presence of co-existing mutations also helps subdivide mIDH gliomas and provides prognostic information. Astrocytomas typically harbor *ATRX* and *TP53* mutations (3, 24), whilst oligodendrogliomas are defined by co-deletions in 1p and 19q and may carry *CIC* and *TERT* mutations (3). Overall survival (OS) is significantly shorter for astrocytomas harboring *CDKN2A/B* deletions in both pediatric and adult patients (21, 71), and therefore the CNS5 classification now upgrades astrocytomas with CDKN2A/B homozygous deletion to WHO grade 4 irrespective of high grade histology features with microvascular proliferation or necrosis (58). The role of *CDKN2A* deletion in patients with oligodendroglioma remains under investigation. The presence of mutations in the *PIK3CA*, *PIK3R1* and amplification of *PDGFRA* and *MYCN* also confer a trend toward poorer survival (72). Genetic alterations involving members of the RB1 pathway, including *CDK4* amplification and *RB1* mutation or homozygous deletion, have also been reported (71).

*IDH* mutation status is an independent predictor of favorable outcomes among adults with glioma (34, 73). In part, this may be due to increased sensitivity to chemotherapy and radiotherapy, as *IDH* mutation does not have the same impact on survival in untreated tumors (74).

### 3.2 Prevalence of IDH-mutant gliomas in pediatric and AYA patients

Given the rarity of *IDH* mutations in pediatric and AYA tumors, there is less detail on their prevalence and clinical impact (75), with most information found in small cohort studies. The prevalence of *IDH* mutations in the pediatric and AYA cohort ranges from 1% to over 50%, with *IDH1* mutations being more common than *IDH2* mutations (**Table 1**). In a cohort of 43 pediatric patients with newly diagnosed WHO grade 3 or 4 gliomas treated on the Children’s Oncology Group ACNS0423 study, *IDH1* mutations were detected in 7 of 43 (16.3%) of children with primary malignant gliomas, and no *IDH2* mutations were identified. Older children and AYA are more likely to have mIDH gliomas. In a large AYA case series including patients aged 15-39.9 by Bennet et al. *IDH* mutations were noted in 57% patients (7) whereas Pollack et al. report *IDH* mutations in 7 of 20 gliomas (35%) from children ≥14 years and 0 of 23 (0%) in younger children (19). The PRecISion Medicine for Children with Cancer (PRISM), trial has reported two patients with mIDH astrocytoma of the 146 patients enrolled with high-risk pediatric brain tumors who had at least 18 months of follow-up (77).

**Table 1:** Frequency of IDH mutations in pediatric and AYA gliomas.

Tumor type	Age range of included patients	Frequency of IDH mutations	Comments	Reference
Historic WHO grade 3/4 glioma	3-21 years	7/43 (16%) *IDH1*m No *IDH2*m		(19)
Non-pilocytic gliomas	Age of “children” not defined	4/73 (5%) *IDH1*m No *IDH2*m	Children with *IDH*-mutated gliomas were older than children with *IDH*-wildtype gliomas	(20)
Glioma *Retrospective review*	0-21 years	78/851 (9.2%) *IDH1*m or *IDH2*m	Patients aged 0-9: 2/378 (0.5%) Patients aged 10-21: 25/277 (9%)	(21)
Grade II and III gliomas	<18 years	4/32 (12.5%) *IDH1*m		(23)
Historic histologic diagnosis pediatric glioblastoma	1-18 years	10/162 (6%) *IDH1*m		(75)
Low grade glioma	<19 years	10/976 (1%) *IDH1* p.R132H mutation	Median age of diagnosis of 15.7 years	(76)
Glioma	15-39.9 years	464/876 (53%)		(7)
High risk pediatric brain tumors	<18 years	2/146	Not exclusively glioma cohort	(77)

### 3.3 Primary mismatch repair-deficient IDH-mutant astrocytomas

Recently, a distinct cohort of patients with IDH-mutant astrocytomas has been recognized with hereditary mismatch repair deficiency (MMR) (78, 79). These patients are unique to those with acquired MMR due to alkylating agents. Instead, primary mismatch repair-deficient IDH-mutant astrocytomas (PMMRDIA) are histologically high grade, often displaying a hypermutant genotype and microsatellite instability (78, 80). These tumors primarily present in younger patients and have worse clinical outcomes compared to other IDH-mutant gliomas (78).

Dodgshun et al. first described six patients with RRD HGG with secondary *IDH1* mutations that clustered with other *IDH1* mutant gliomas on methylation profiling (79). However, while other mIDH gliomas demonstrated a CpG Island Methylator Phenotype (CIMP), these six tumors with secondary *IDH1* mutations displayed the inverse pattern of methylation disturbance; a CpG Island Demethylator Phenotype (CIDP) in keeping with other RRD HGG. These findings suggest that the CIDP phenotype generated by RRD glioma is unable to be overcome by secondary IDH mutations.

In a study by Suwala et al., samples from 32 patients with IDH-mutant gliomas and proven or suspected primary MMR deficiency were sequenced. Patients were clinically diagnosed with Lynch syndrome or Constitutional Mismatch Repair Deficiency Syndrome and/or had germline mutations in DNA mismatch repair genes (*MLH1*, *MSH6*, *MSH2*) in all but one case. Results demonstrated a distinct DNA methylation profile which clustered separately from other IDH-mutant glioma subtypes including those with acquired MMR deficiency on t-distributed stochastic neighbor embedded (t-SNE) plots. Tumors displayed a higher proportion (60%) of unmethylated MGMT promoter compared to other IDH-mutant gliomas. Frequent inactivation of *TP53*, *RB1*, *ATRX* and activation of the *RTK*/*PI3K*/*AKT* pathway was also present. The OS of this cohort of patients was significantly worse, with a mean OS of only 15 months despite treatment with surgery, radiation and chemotherapy. Pediatric and AYA patients diagnosed with a mIDH glioma warrant screening for MMRD.

## 4 The current standard of care

Recommended management algorithms for mIDH glioma have been defined by several groups including the American Society of Clinical Oncology (ASCO) (24), the Society for Neuro-Oncology (SNO) (2), and the European Association of Neuro-Oncology (EANO) (25). These algorithms incorporate surgery and outcomes from randomized clinical trials on the use of radiotherapy and/or chemotherapy. It is important to consider that most data and recommendations pertain to adult patients with potential for underrepresentation of the AYA group and limited trials conducted in the pediatric setting.

### 4.1 Role of surgery

The benefits of safe maximal resection in adults with glioma were well-established prior to the era of molecular characterization. These results have been recapitulated in the modern diagnostics era by Wijenga et al. (81) with a retrospective re-analysis of adult tumor subtypes applying new molecular classification. This study demonstrated that for mIDH astrocytomas, each 1cm^3^ increase in postoperative volume of disease results in poorer OS, hazard ratio (HR) of 1.01 (p<0.0001). However, the impact of extent of upfront resection is less clear in pediatric patients. In a multi-institutional retrospective analysis of 78 pediatric patients with *IDH1/2* mutated gliomas including 45 patients with low grade astrocytoma, the 5Y PFS of patients with gross total resection (GTR) was 45.7% with median PFS of 4.4 years compared to 5Y PFS of patients with subtotal resection (STR) of biopsy of 41% with median PFS of 4.7 years (21). There was no statistically significant difference in OS in this cohort. With only 13 patients in the cohort of low-grade oligodendrogliomas in this study, there was no statistically significant differences in PFS or OS for upfront GTR versus STR or biopsy. The discrepancies in outcomes compared to adults may be reflective of small sample size.

In adults, repeat craniotomy and safe resection is feasible for patients with recurrent LGG (82). Re-operation in eloquent or near eloquent brain areas are not associated with higher risk of neurological sequelae compared to initial surgery (83). Up to 50% of patients can achieve a gross total resection at time of recurrence (84). Mounting evidence demonstrates that there is survival benefit to near total or gross resection at time of recurrence (83–85). Thus, standard of care is moving towards aggressive safe early resection at recurrence.

### 4.2 Adjuvant therapy

In adult IDH-mutant grade 2 gliomas a “watch and wait” approach may be taken for “low-risk” patients, historically defined as younger patients under 40 years with GTR (2, 86). For “high-risk” patients, postoperative combination radiotherapy and sequential chemotherapy is standard of care (2, 24, 25). The most common chemotherapy regimens are procarbazine, lomustine and vincristine (PCV) or temozolomide (TMZ). All cooperative groups recommend radiotherapy and sequential chemotherapy for patients with Grade 2 IDH-mutant gliomas aged over 40 years and with STR (2, 24, 25). Patients with Grade 3 oligodendrogliomas with 1p/19q co-deletion should receive radiotherapy with PCV (2, 24, 25). Those with Grade 3 astrocytomas and no 1p/19q co-deletion receive radiotherapy with adjuvant TMZ (2, 24, 25).

TMZ is an oral alkylating agent that results in the methylation of DNA at the O^6^-guanine, as well as N^7^-guanine and N^3^-adenine residues (87). Methylation of O^6^-guanine results in the O^6^-methylgaunine (O^6^-meg) lesion. O^6^-meg lesions are repaired by O^6^-methylguanine DNA methyltransferase (MGMT), a process that is dependent on the number of MGMT molecules per cell and rate of MGMT regeneration (88). Unrepaired O^6^-meg lesions incorrectly pairs with thymine during DNA replication instead of cytosine, triggering the DNA mismatch repair (MMR) system and repeated cycles of double-stranded breakages in a process called futile cycling (87). The accumulation of these DNA strand breaks eventually results in cell cycle arrest and activation of apoptosis. Thus, cytotoxicity of TMZ is dependent on an intact MMR pathway and low levels of MGMT.

The CATNON trial (89), a phase 3 randomized controlled trial, established the use of adjuvant rather than concurrent TMZ with radiotherapy on 1p/19q non-co-deleted anaplastic gliomas, particularly those with IDH mutations. The CATNON trial used a 2 x 2 factorial design, enrolling 751 patients to receive either radiotherapy alone, or radiotherapy with concurrent and/or adjuvant TMZ. Adjuvant TMZ showed significant improvement in OS (median OS 82.3 months, 5Y OS 56% vs median OS 46.9 months, 5Y OS 44% in control arm). The second interim analysis did not demonstrate a statistically significant benefit of concurrent TMZ compared to radiotherapy alone. TMZ was generally well tolerated with the most common Grade 3 and 4 TRAE being related to hematological side effects, particularly thrombocytopenia and neutropenia.

Importantly, this study holds significant relevance for IDH-mutant gliomas. Mutation status was analyzed in 671 of the 751 patients, with 436 harboring *IDH1* or *IDH2* mutations, evenly distributed across the four arms of the study. Overall, *IDH1/IDH2* mutation status had a significant impact on survival with median OS 19.9 months in the wild-type group vs 98.4 months in the IDH-mutant group (HR 0.14, p<0.0001). Significantly, in patients with *IDH1* and *IDH2* wild-type tumors, neither concurrent nor adjuvant TMZ improved OS compared with radiotherapy alone, whereas IDH-mutated tumors had significantly improved OS with the addition of adjuvant TMZ. Median PFS in IDH-mutant patients treated with any TMZ was 77.0 months compared to 34.2 months in patients treated with radiotherapy alone (p<0.0001).

The results of the CATNON trial suggest that there is a crucial role for adjuvant TMZ in adult IDH-mutant LGG and this is currently recommended as standard of care in the settings outlined above. However, in the pediatric and AYA population TMZ-induced hypermutation and acquired resistance need to be considered given the potential for prolonged survival and ongoing exposure to alkylating agents. TMZ-resistance may occur through the mutagenic action of TMZ on DNA repair genes resulting in acquired deficiencies in MMR (90). Loss of MMR function leads to ongoing mispairing of guanine with thymine; however, unrepaired DNA damage is no longer detected and cells are unable to activate apoptosis (91). In the absence of MGMT-mediated repair and intact MMR, cells incur a large number of G:C>A:T transitions throughout the genome upon DNA replication, including thousands in coding regions (87). This mutational signature is known as single base substitution (SBS) signature 11 and is commonly seen in hypermutated gliomas after exposure to alkylating agents (92). The acquisition of mutations that inactivate the MMR pathway and continued TMZ exposure results in hypermutation. Despite these considerations, TMZ remains widely used in pediatric HGG.

Another recent trial, the CODEL trial, was initially designed to compare the efficacy of radiotherapy alone, radiotherapy + TMZ, or TMZ monotherapy in patients with 1p/19q codeleted grade 3 oligodendroglioma (93). Initial analysis demonstrated a significantly shorter PFS in patients receiving TMZ monotherapy compared to RT (HR 3.12, p=0.014). Of the patients included that were evaluable for IDH mutation status, 30 (86%) were IDH mutated. Given the results, the trial has been redesigned to compare adjuvant radiotherapy + TMZ and radiotherapy + PCV among patients with grade 2 and 3 oligodendroglioma. The trial is ongoing with results awaited. However, recent results from the French POLA network demonstrate that in a cohort of 306 patients with grade 3 oligodendroglioma, radiotherapy + PCV was associated with significantly improved 5-year and 10-year OS compared to radiotherapy + TMZ (5Y OS 89% PCV vs 75% TMZ, p=0.0014; 10Y OS 73% PCV vs 60% TMZ, p=0.0003) (94).

## 5 Mutant IDH inhibitors

Surgical resection, chemotherapy and radiation therapy are associated with treatment-related toxicities including long term neurocognitive disorders (1). Targeted therapies are clearly of emerging interest to avoid long-term toxicities particularly in the pediatric and AYA cohort.

IDH inhibitors reduce D2-HG concentrations in tumor xenograft models (95) and in clinical pharmacology studies (26, 96, 97). Inhibition of mutant *IDH* in tumor cells, and the associated reduction in D2-HG production, can restore normal cellular differentiation and provide therapeutic benefit in cancers harboring IDH mutations (42). There are several *IDH* inhibitors currently under clinical investigation with biologic activity, good tolerance, and evidence of clinical activity. Studies have demonstrated the safety and feasibility of these agents in patients with glioma, summarized in **Table 2**.

**Table 2:** Trials of IDH inhibitors in patients with glioma.

Reference	No. of patients	Age range	Disease type	Phase of trial	Single agent/combined	Grade ≥ 3 treatment related adverse events (TRAE)	Efficacy data	Enhancing vs non-enhancing
Ivosidenib								
Mellinghoff, Ellingson et al. (2020) (98)	66	21-71y	Relapsed/refractory mIDH1 Glioblastoma n = 12 LGG n = 54	Phase I, multicenter, open-label study	Single agent	Neutropenia, weight loss, hyponatremia, arthralgia (n = 1, 1.5% each)	PR n = 1 (1.5%) SD = 44 (66.7%) PD n = 21 (31.8%)	Median PFS 13.6 months enhancing, 1.4 months non-enhancing
Puri, Shi et al., 2022)	12	26-62y	WHO grade 2/3 IDH-mutated astrocytoma and oligodendrogliomas	Retrospective review	Off-label ivosidenib	No comment	PFS 12 months 88% (n=8)	No difference
Olutasidenib								
de la Fuente, Colman et al. (2023) (99)	26	IQR 40-49y	R/R solid tumor or glioma	Phase 1b/2	Combined	Nausea 14/26 (54%), fatigue 13/26 (50%), ALT increased 8/26 (31%), diarrhea 8/26 (31%), headache 8/26 (31%), constipation 7/26 (27%)	ORR + SD: 12/25 (48%) of 25	Both responders enhancing tumors at baseline
BAY1436032								
Wick, Bahr et al. (2021) (100)	81 (39 in low grade glioma cohort)	19-81y	*m*IDH1 solid tumors: LGG n = 39 Glioblastoma n = 16 Intrahepatic Cholangiocarcinoma n= 16 Other tumor types n= 10	Phase I	Single agent	12% (6/52) TRAE grade ≥3 (incl. grade 4 lipase increase)	In LGG cohort: CR 3% (1/35), PR 9% (3/35), ORR 11% (4/35)	33/35 patients had enhancing lesion
Safusidenib								
Natsume, Arakawa et al. (2023) (97)	47	28-77y	Recurrent/progressive IDH1-mutant (R132) glioma	Multicenter, open-label, dose-escalation, phase I study	125-1400 mg twice daily	Neutropenia (n = 6 12.8%), diarrhea (n = 2, 4.3%), arthralgia (n = 1, 2.1%), headache (n = 1, 2.1%), raised liver function tests (n = 5 10.7%) and hypophosphatemia (n= 2 4.3%).	ORR = 17.1% (enhancing tumors), 33.3% (non-enhancing tumors)	ORR greater for non-enhancing tumors (33.3% ORR vs 17.1%)
IDH305								
DiNardo, Schimmer et al. (2016) (101)	81 in total, 32 glioma patient	29-85y	Glioma	Phase I	Single agent dose escalation	Elevated bilirubin 4/81 (49%), Elevated lipase 1/81 (1.2%), rash 1/81 (1.2%)	Not reported	N/A
Vorasidenib								
Mellinghoff, Penas-Prado et al. (2021) (102)	93	16-89y	mIDH1/2 solid tumors, including 52 patients with glioma that had recurred or progressed following standard therapy	Open label Phase 1	Vorasidenib orally, once daily	Dose-limiting toxicities of elevated transaminases occurred at doses ≥100 mg and were reversible	Non-enhancing gliomas: ORR = 18%, median PFS 36.8 months. Enhancing gliomas: No radiographic response; median PFS 3.6 months	Outcomes for non-enhancing gliomas superior to enhancing gliomas
Mellinghoff, van den Bent et al. (2023) (26)	331 (168 vorasidenib, 163 placebo)	16-71y	Residual or recurrent grade 2 IDH-mutant glioma who had undergone no previous treatment other than surgery	Double-blind, phase 3 trial	Oral vorasidenib (40 mg once daily) or matched placebo	Grade ≥3 AE 22.8% vorasidenib, 13.5% placebo. Grade ≥3 AE increased ALT in 9.6%	Median PFS 27.7 months (vorasidenib) vs. 11.1 months (placebo)	Excluded enhancing lesions

The United States Food and Drug Administration (FDA) and the Australian Therapeutic Goods Administration (TGA) recently approved one such agent, vorasidenib, for adult and pediatric patients 12 years and older with grade 2 astrocytoma or oligodendroglioma with a susceptible IDH1 or IDH2 mutation (103).

### 5.1 IDH1 specific

#### 5.1.1 Ivosidenib (AG120)

Ivosidenib is an oral, potent, highly specific targeted small-molecule inhibitor of mutant *IDH1* (1, 13). Preclinical data shows that ivosidenib inhibits invasion and migration of *IDH1* mutated chondrosarcoma cell lines (104). Pharmacokinetic analysis showed that ivosidenib was detectable in the brain-tumor tissue, suggesting the molecule is able to cross the blood-brain barrier (105). Mouse xenograft models of human mIDH1-R132H glioma show strong inhibition of D2-HG production in brain tumor samples (105). Furthermore, plasma D2-HG levels decreased in adults treated with ivosidenib (106).

Clinical studies using ivosidenib have been conducted in adults with CNS tumors (98), cholangiocarcinoma (107–109), chondrosarcoma (106), and myeloid malignancies (96, 110–112), either as a single agent or in combination with chemotherapy (**Supplementary Table 1**). The most common grade ≥ 3 treatment related adverse events (TRAE) include myelosuppression, ascites, and QT prolongation.

#### 5.1.2 Olutasidenib (FT-2102)

Olutasidenib (FT-2102) is a potent, selective, oral, small-molecule inhibitor of mutant *IDH1* that entered clinical development in 2016 (17, 42, 113). Olutasidenib is a blood brain barrier penetrant (95, 114) quinolinone-based non-competitive inhibitor of mutant *IDH1* that binds to an allosteric site, a hydrophobic pocket near the IDH1 homodimer interface (17, 115).

In adult clinical trials in myeloid malignancies (17, 42, 116) and relapsed/refractory solid tumors (including gliomas) (99), the drug has been well tolerated with most common serious (Grade 3 and above) TRAE include hepatic transaminitis and myelosuppression (**Supplementary Table 2**). Other common TRAE included nausea, fatigue, diarrhea, constipation, and headache (99). Olutasidenib was granted FDA approval to treat patients with relapsed/refractory *IDH1* mutant AML on 1 December 2022 (42, 113).

In a phase 1b/2 trial enrolling 26 adult patients with relapsed/refractory WHO Grade 3 and 4 *IDH1* R132X-mutant glioma, olutasidenib resulted in a disease control rate (objective response plus stable disease) of 48% (99). Best response was partial response, achieved in two (8%) patients and eight patients (32%) had stable disease for at least four months as per Response Assessment in Neuro-Oncology (RANO) response criteria. These results suggest olutasidenib demonstrates preliminary evidence of clinical activity in a heavily pretreated population.

#### 5.1.3 BAY1436032

BAY1436032 is an oral small-molecule inhibitor of R132-mutant *IDH1* that is active in preclinical models of mIDH1 cancer (117). BAY 1436032 strongly reduces D2-HG levels in cells carrying *IDH1*-R132H, -R132C, -R132G, -R132S and -R132L mutations, with a median maximal reduction of plasma R-2-hydroxyglutarate levels of 76% (100, 118). BAY 1436032 partially crosses the blood brain barrier; maximal intraparenchymal BAY 1436032 concentration in mouse brain amounted to 38% of that in plasma levels (118).

BAY1436032 has been studied in adults with solid tumors (100) and AML (119) (**Supplementary Table 3**). The most common serious TRAE include raised lipase levels and myelosuppression.

#### 5.1.4 Safusidenib (DS-1001B/AB-218)

Safusidenib (DS-1001b/AB-218) is an orally available, brain-penetrant, selective inhibitor of R132-mutant *IDH1* that has shown efficacy in preclinical models of glioma (49, 120). It has been studied in adults with recurrent or progressive mIDH glioma (**Supplementary Table 4**) (97). Grade ≥ 3 TRAE related to DS-1001 included neutropenia and hepatotoxicity. Studies of Safusidenib in patients with IDH1-mutated WHO glioma are ongoing (NCT04458272, NCT05303519 and NCT05577416).

#### 5.1.5 IDH305

IDH305 is a potent and selective mutant IDH1 inhibitor that has demonstrated brain exposure in rodents, D2-HG reduction, and efficacy in a patient-derived *IDH1* mutant xenograft tumor model (121). It has been studied in adults with CNS tumors and myeloid malignancy, identifying hepatotoxicity, tumor lysis syndrome and differentiation syndrome as the most common serious TRAE (**Supplementary Table 5**) (101, 122).

### 5.2 IDH2 specific

#### 5.2.1 Enasidenib (AG221)

Enasidenib is a small molecule inhibitor of the IDH2 enzyme. In preclinical studies, enasidenib decreased total serum D2-HG by more than 90%, reduced abnormal histone hypermethylation, and restored myeloid differentiation (96, 123, 124). In patients with *IDH2*-mutated AML, enasidenib reduced serum D2-HG levels resulting in increased percentages of mature myeloid cells in the bone marrow (112).

Enasidenib has been studied in adults with AML (**Supplementary Table 6**) (96, 125, 126). The most common serious adverse reaction was QT prolongation, hepatotoxicity, and myelosuppression.

There is no evidence that enasidenib crosses the blood brain barrier. *IDH*2 mutations are less prevalent in gliomas, rendering an IDH2 inhibitor less likely to be beneficial in this cohort.

### 5.3 Combined IDH1 and IDH2 inhibitors

#### 5.3.1 Vorasidenib (AG-881)

Vorasidenib is an oral drug pan inhibitor of both mutant IDH1 and IDH2, shown to penetrate the mouse brain and reduce D2-HG production by over 97% in an orthotopically engrafted patient-derived xenograft (PDX) (127, 128). A recent study revealed that isoform switching from mIDH1 to mIDH2 or *vice versa* may represent a mechanism of acquired resistance, sparking interest in the use of pan-inhibitors (129, 130).

Vorasedinib has been studied in adults with solid tumors (26) and hematologic malignancy (130) (**Supplementary Table 7**). Vorasidenib is well tolerated with grade 3 or higher TRAE occurring in less than half of patients (26). The most common adverse Grade 3 or higher TRAE include hepatic transaminitis, diarrhea, and gastrointestinal hemorrhage. A small number of patients require dose reduction or cessation due to adverse effects (26).

Mellinghoff et al. (131) report the use of vorasidenib or ivosidenib for *IDH*-R132H mutant LGG in a perioperative phase 1 trial. In this study, patients received pre-operative vorasidenib or ivosidenib and continued treatment post-surgery until disease progression or unacceptable toxicity. Tissue analysis was performed from 40/49 patients, demonstrating a reduction in concentration of D-2-hydroxyglutarate (D2-HG), the metabolic product of mIDH enzymes, by 92.6% following vorasidenib and 91.1% following ivosidenib compared to patients receiving placebo. Both drugs were well tolerated without any surgical delays. In the vorasidenib group, 7/24 (29.2%) experienced grade 3 or higher TRAE including brain abscess, tooth infection, aphasia, brain edema, hydrocephalus, alanine aminotransferase (ALT) increase, anemia, hyperglycemia, and hypophosphatemia. Rate of grade 3 TRAE was similar in the ivosidenib group with 6/25 (24%) experiencing hyponatremia, leukopenia, subdural hematoma, invasive ductal breast carcinoma, brain edema, brain injury, hemiparesis, syncope, mental status change and pneumothorax.

Anti-tumor effect was assessed with objective response rate (ORR) in the group treated with 10mg daily vorasidenib 10% (1/10), and 50mg daily vorasidenib 42.9% (6/14). For ivosidenib, those treated with 250mg twice daily had ORR 12.5% (1/8) compared with 35.7% (5/14) treated with 500mg daily. Based on these preliminary antitumor activity and enzyme inhibition findings, vorasidenib 50mg daily was initially carried forward to the global phase 3 INDIGO study in grade 2 mIDH non-enhancing glioma (26). A coated-tablet formulation equivalent to 40mg daily has since been introduced.

The pivotal double-blinded, phase 3 INDIGO study (26) evaluated vorasidenib in adult patients with residual or recurrent grade 2 non-enhancing IDH-mutant gliomas. Of 331 patients, 168 patients were randomized to receive vorasidenib 40mg once daily. Vorasidenib significantly improved imaging-based PFS compared to placebo (median PFS 27.7 months vs 11.1 months, HR 0.39, p<0.001). Additionally, vorasidenib delayed the need for further interventions (HR 0.26, p<0.001). At a median follow-up of 14.0 months, 131 were continuing to receive vorasidenib within the intervention group. The impact on OS will take years to elucidate but will be likely confounded by the crossover design (132). Furthermore, although the eligibility criteria included patients aged 12 years and over, the youngest patient enrolled was 16 years of age and no patients under 18 years of age were randomized to the intervention arm. AYA patients were also underrepresented in this cohort with a mean age of 40.5 years.

### 5.4 Introducing IDH inhibitors into standard of care treatment

Understanding of the biological impact of IDH inhibitors in glioma is evolving. Preclinical modelling in mIDH glioma is complicated by the indolent nature of the tumors which makes generation of patient-derived cell lines and xenograft models difficult (49, 133). However, using a combination of RNA single-cell analysis, bulk RNA-sequencing analysis and *in vitro* modelling, Spitzer et al. were able to demonstrate differentiation of oligodendrogliomas to an “astrocytoma-like” phenotype with decreased cell cycle expression, thus resulting in less proliferation, in patients treated with vorasidenib (134).

The specific role of IDH mutations in driving tumor growth and aggressiveness at the time of recurrence is also unclear. Recurrent tumors are more likely to display a hypermutation phenotype and genome-wide loss of DNA methylation (135, 136). Several recent studies also confirm the present of copy number alterations, particularly *CDKN2A/B* loss and *CDK4* amplification, clinically conferring poorer prognosis in IDH-mutant astrocytomas (137–141). However, the effect of *CDKN2A/B* deletion in oligodendroglioma is less clear (142). These findings may explain the clinical data demonstrating poorer survival outcomes of high-grade mIDH glioma patients treated at recurrence with mIDH inhibitors (143).

The role of IDH inhibitors in standard of care for IDH-mutant gliomas is yet to be determined. Overall, studies suggest superior activity in patients with non-enhancing tumors compared with enhancing tumors (97, 98, 100, 102), with enhancement typically indicating transformation of LGG to higher grades (98). The suggestion based on the above trials would be that these inhibitors may be useful in the early, indolent course of disease (133). However, many questions remain including the efficacy of IDH inhibitors compared to current standard of care radiotherapy and chemotherapy regimens.

### 5.5 Emerging mechanisms of resistance

There is a lack of current understanding of how prolonged use of IDH inhibitors alters the biology of mIDH gliomas, leading to resistance (133). *IDH* inhibitor resistance has been reported in adults with AML through *trans* or *cis* dimer-interface mutations (144), receptor tyrosine kinase (RTK) pathway mutations (145) or second site mutations (146, 147). In adults with cholangiocarcinoma resistance mechanisms include a second IDH mutation (129, 148). Understanding the mechanism of resistance is important to guide salvage treatment options and to identify other potential targets.

### 5.6 Combination studies

Several reports and a recently published clinical trial by the International Replication Repair Deficiency Consortium (IRRDC) demonstrating durable responses and prolonged survival in patients with glioma and high mutational burden (149, 150). In contrast, in Suwala’s study of patients with PMMRDIA, limited effect was seen in three patients treated with immune checkpoint inhibitors (ICI) (78). A subsequent IRRDC study of mIDH RRD HGG confirmed lower PFS in mIDH RRD HGG compared to sporadic mIDH HGG treated with ICI monotherapy (151).

The lack of efficacy in this cohort is thought to be attributed to the immune suppressive effects of the IDH mutation on the tumor microenvironment (80). IDH mutations result in down-regulation of genes involved in immune activation of cancer cells likely occurring either by a direct metabolic inhibitory effect of D2-HG or through epigenetic reprogramming by hypermethylation of promotors of immune-related genes (47). Hence, D2-HG strongly represses T-cell activity, acting as a protective mechanism for tumor cells by evading the immune system. Furthermore, hypermethylation has been implicated in the downregulation of immune checkpoints such as PD-L1 and CTLA4 (78, 152).

The combination of IDH inhibitors with ICI is therefore promising, specifically for PMMRDIA where novel therapeutic strategies are required. In this combination, IDH mutation driven immunosuppression could be reversed with IDH inhibitor treatment, enhancing immune checkpoint blockade. In the clinic, Das et al. have reported favorable responses for mIDH-RRD-HGG receiving combination ICI and IDH inhibitor therapy with prolonged survival at 12 months compared to those without IDH mutations (151). To test this further, a number of clinical trials are ongoing including a phase 2 trial combining nivolumab with ivosidenib in patients with advanced IDH-mutant solid tumors (NCT04056910) as well as a phase 1 trial of pembrolizumab and vorasidenib in patients with relapsed/refractory Grade 2 and 3 mIDH glioma (NCT0584622).

## 6 Need for trials for IDH-mutant LGG in children, adolescents and young adults

As outlined above, pediatric IDH-mutant LGG are rare, although with increased access to advanced genomic testing, these tumors are being identified more readily (21). There remains a paucity of data to describe their natural history, treatment strategies and outcomes. Specifically, the biological characteristics and behavior compared to adult IDH-mutant LGG remains unclear (21). Malignant transformation in the adult cohort is well documented (153) and an aggressive upfront approach is warranted. In comparison to other pediatric/AYA LGG patients, mIDH tumors appear to have a higher risk for malignant transformation (21, 76); however is it unclear whether IDH mutations confer the same prognostic significance as in the adult cohort.

These considerations have critical treatment implications as approaches vary significantly amongst institutions with no established standard of care. Radiotherapy may be delayed or avoided; in the retrospective study by Yeo et al. only 22/76 (28.9%) of patients received upfront radiotherapy (21). In the adult setting, radiotherapy is a cornerstone to the treatment of gliomas and attempts to de-intensify treatment post resection to chemotherapy alone have so far proved unsatisfactory (93, 132). Significant concerns regarding toxicities in children are potential reasons to consider deferral of radiotherapy (132, 154). In particular, CNS directed radiotherapy has neurocognitive sequelae, including decline in cognition, processing speed, fine motor skills, verbal fluency, and delayed attention (154). Endocrinopathies can results in issues with growth and puberty (154). There is an increased risk of late neurovascular events such as stroke (155). However, radiotherapy, particularly in the setting of STR or recurrent/progressive disease, plays an important role in achieving local control and prolonging PFS (154). Therefore, the optimal timing and modality is important to clarify.

The use of novel targeted therapies is an attractive option to prevent long-term toxicities in pediatrics. However, access to IDH inhibitors for pediatric patients with IDH-mutant tumors is difficult. Currently, ivosidenib has available pediatric dosing based on a COG Phase II MATCH trial for *IDH1* mutant tumors. However, this trial was closed early prior to meeting study endpoints due to slow accrual (NCT04195555). Although vorasidenib has been approved by the FDA, there is no published dosing for children under 12 years. An upcoming CONNECT trial (NCT06161974) will assess the combination of olutasidenib (FT-2102) and temozolomide for pediatric IDH-mutant HGG. The sparsity of clinical trials leaves many questions left to be answered such as optimal timing and duration of therapy.

Furthermore, the long-term impact of these novel targeted therapies is unknown. Pleasingly, health related quality of life (HrQOL) and neurocognitive outcomes assessed for adult patients enrolled in the INDIGO trial did not demonstrate any clinically meaningful deterioration from baseline at any timepoint compared to placebo (156). While these preliminary data suggest that the neurocognitive effects of these agents are acceptable in the adult population, this needs to be weighed against the impact of potential tumor progression, a key driver of cognitive decline (132). Given the skewed burden of IDH-mutant gliomas to older children and adolescents, fertility is also an important consideration given the unknown impact on fertility, pregnancies, and the fetus (132).

The role of IDH inhibitors in standard of care for IDH-mutant gliomas is yet to be determined. Overall, studies suggest superior activity in patients with non-enhancing tumors compared with enhancing tumors (97, 98, 100, 102), with enhancement typically indicating transformation of LGG to higher grades (98). The suggestion based on the above trials would be that these inhibitors may be useful in the early, indolent course of disease (133). However, many questions remain including the efficacy of IDH inhibitors compared to current standard of care radiotherapy and chemotherapy regimens.

We suggest that by early treatment with these inhibitors we may change the natural history of eventual progression to higher risk disease and eventual death of these patients. By intercepting the physiologic changes to the tumor and its microenvironment, we propose we completely alter the disease course and prevent the eventual development of high grade glioma.

## 7 Conclusion

While several advances have been made in the field of adult IDH-mutant LGG, much is still left to be elucidated in the pediatric and AYA setting. Increased access to next-generation sequencing has resulted in the increased recognition of these tumors affecting older children and adolescents. Tumor biology in this group is unclear and the impact of available therapies commonly used in the adult setting need to be carefully considered. Temozolomide, used frequently in the adult setting for its favorable toxicity profile, has a clear risk of hypermutation whilst IDH inhibitors have not been tested in the pediatric CNS tumor population and need further study to determine impact on tumor biology and efficacy at recurrence. A small but significant proportion of pediatric patients with mIDH glioma will have PMMRDIA, with poorer survival outcomes and therefore this must be considered at diagnosis in this age group. Current practice remains variable with no standard of care defined, demonstrating an urgent need for pediatric-specific clinical trials.

## Data Availability

The original contributions presented in the study are included in the article/**Supplementary Material**. Further inquiries can be directed to the corresponding authors.
