# Supplementary material for: IDH-mutant gliomas in children and adolescents - from biology to clinical trials
Source: Front Oncol. 2025 Jan 6;14:1515538. doi: 10.3389/fonc.2024.1515538 (PMC11773619; doi:10.3389/fonc.2024.1515538)
Supplement: Supplementary file 1 [file Table1.docx]

# Supplementary Tables

Supplementary Table 1: Ivosidenib in clinical trials

| **Reference** | **Number of Patients** | **Disease Type** | **Phase of Trial** | **Single agent/ combined** | **Adverse events** | **Efficacy Data** |
| --- | --- | --- | --- | --- | --- | --- |
| DiNardo, Stein et al (2018)  (116) | 179 | IDH1-mutated AML - relapsed or refractory | Phase 1 dose-escalation and dose-expansion | 500 mg of ivosidenib daily | Treatment-related grade ≥ 3: prolonged QT interval (7.8%), IDH differentiation syndrome (3.9%), anemia (2.2%), thrombocytopenia (3.4%), and leucocytosis (1.7%) | CR+CRi = 30.4%  CR = 21.6% ORR = 41.6% |
| Lowry, Burris et al (2019) (111) | 73 patients | mIDH1-cholangiocarcinoma | Phase I dose-escalation | Single agent | Grade ≥ 3 events: ascites (5%) and anaemia (4%) | PR n = 4 (5%) |
| Abou-Alfa, Macarulla et al (2020)  (112)  Zhu, Macarulla et al (2021)  (113) | 185 patients | IDH1-mutant cholangiocarcinoma (refractory) | Phase III | Ivosidenib 500 mg or matched placebo once daily | Grade ≥ 3 events: ascites (7%) | PFS ivosidenib vs placebo median 2·7 months [95% CI 1·6–4·2] *vs* 1·4 months [1·4–1·6]. Hazard ratio 0·37; 95% CI 0·25–0·54 |
| Mellinghoff, Ellingson et al (2020)  (102) | 66 patients | Relapsed/refractory mIDH1  Glioblastoma n = 12  LGG n = 54 | Phase I, multicentre, open-label study | Single agent | Grade ≥ 3 events: neutropenia, weight loss, hyponatremia, arthralgia (n = 1, 1.5% each) | PR n = 1 (1.5%)  SD = 44 (66.7%)  PD n = 21 (31.8%) |
| Roboz, DiNardo et al (2020) (114) | 34  Dose-escalation phase n = 9  Dose-expansion phase n = 25 | Newly diagnosed mIDH1  AML | Phase 1, multicentre, open-label, dose-escalation, and dose-expansion study | 500 mg once daily | Grade ≥3 AE: 79%  Differentiation Syndrome (n = 3; 9%), ECG QT-prolonged, febrile neutropenia, diarrhoea (n = 2; 6% each) | CR+CRh rate: 42.4%  CR rate: 30.3% |
| Tap, Villalobos et al (2020)  (110) | 21 patients | Advanced chondrosarcoma | Phase I multicentre open-label dose-escalation and expansion study | Ivosidenib 100 mg twice daily to 1,200 mg once daily | Grade ≥ 3 events: 57%  Treatment related: n = 1 (hypophosphatemia) | Median PFS 5.6 months (95% CI, 1.9 to 7.4 months); SD: 11/21 (52%) |
| DiNardo, Stein et al (2021)  (115) | 23 patients | Newly diagnosed mIDH1 AML ineligible for intensive induction chemotherapy | Open-label, multicentre, phase Ib trial | Ivosidenib plus azacitidine | Treatment-related grade ≥ 3: neutropenia (22%), anaemia (13%), thrombocytopenia (13%), and QT prolongation (13%). | ORR = 78.3% (18/23)  CR rate: 60.9% (14/23) |
| Stein, DiNardo et al (2021)  (99) | 60 | mIDH1 newly diagnosed AML | Phase 1, multicentre, open-label study enrolled | Ivosidenib in combination with cytarabine and either daunorubicin idarubicin | Treatment-related grade ≥ 3: QT prolongation (10%) | Cri = 55% or CRp (CR/CRi/ CRp) = 72% at end of induction |
| Puri, Shi et al (2022) Puri, Shi et al. 2022) | 12 | WHO grade 2/3 IDH-mutated astrocytoma and oligodendrogliomas | Retrospective review | Off-label ivosidenib | Retrospective review | Retrospective review |

Supplementary Table 2: Olutasidenib in clinical trials

| **Reference** | **Number of Patients** | **Disease Type** | **Phase of Trial** | **Single agent/ combined** | **Toxicity** | **Efficacy** |
| --- | --- | --- | --- | --- | --- | --- |
| De Botton, Fenaux et al (2023)  (101) | 153 | AML | Phase1/2 | Both | Grade ≥ 3 events hepatotoxicity (13%) |  |
| de la Fuente, Colman et al (2023)  (103) | 26 | R/R solid tumor or glioma | Phase 1b/2 | Combined | SAEs: 11/26 (42%) TEAEs: nausea 14/26 (54%), fatigue 13/26 (50%), ALT increased 8/26 (31%), diarrhoea 8/26 (31%), headache 8/26 (31%), constipation 7/26 (27%) | ORR + SD: 12/25 (48%) of 25 |
| Watts, Baer et al (2023) (17) | 78 | AML or intermediate, high, or very high-risk myelodysplastic syndrome harbouring mutant IDH1 | Phase 1/2, multicentre, open-label clinical trial | Monotherapy (n=32) or in combination with azacitidine (n=46) | Grade ≥ 3 TEAE monotherapy: thrombocytopenia 9/32 (28%), febrile neutropenia 7/32 (22%) and anaemia 7/32 (22%)  Grade ≥ 3 TEAE combination therapy: thrombocytopenia 19/46 (41%), febrile neutropenia 13/46 (28%), anaemia 9/46 (20%). | R/r AML: 41% ORR monotherapy, 46% combination therapy. Treatment-naive patients AML: ORR monotherapy 25% , combination therapy 77%. |
| Cortes, Jonas et al (2024)  (120) | 18 patients (12 of these patients previously described in (101) | Relapsed/ refractory/CRi (CR with incomplete hematologic recovery) AML | Phase 2 | Mostly single agent. Two of 18 patients received the combination of olutasidenib and azacitidine | Grade ≥ 3 TEAE: decreased RBC count (33.3%), decreased neutrophil count (22.2%), febrile neutropenia (16.7%), and pneumonia (16.7%)*  N = 5 AE resulting in study drug withdrawal (Grade 3-4 increased LFTs (n=3), fatal enterococcal bacteraemia, and fatal COVID-19) | CRc rate: 43.8% |

*Note some of these patients treated with combination therapy

Supplementary Table 3: BAY1436032 in clinical trials

| **Reference** | **Number of Patients** | **Disease Type** | **Phase of Trial** | **Treatment** | **Adverse effects** | **Efficacy Data** |
| --- | --- | --- | --- | --- | --- | --- |
| Heuser, Palmisiano et al (2020)  (123) | 27 | AML | Phase I | Single agent | 26% (7/27) TEAE of ≥grade 3 (incl. grade 4 anaemia, ileus, neutropenia, thrombocytopenia, and sepsis (grade 4). | OR 15% (4/27) |
| Wck, Bahr et al (2021)  (104) | 52 | *m*IDH1 solid tumors LGG n = 25  Glioblastoma n = 13 intrahepatic Cholangiocarcinoma n= 7  Other tumor types n= 7 | Phase I | Single agent | 12% (6/52) TEAE grade ≥3 (incl. grade 4 lipase increase) | OR = 6%  SD 41%  In LGG cohort: best response CR in 3% (1/35) and PR in 9% (3/35)  ORR 11% (4/35) |

Supplementary Table 4: DS-1001 in clinical trials

| **Reference** | **Number of Patients** | **Disease Type** | **Phase of Trial** | **Treatment** | **Adverse effects** | **Efficacy Data** |
| --- | --- | --- | --- | --- | --- | --- |
| Natsume, Arakawa et al (2023)  (100) | 47 | Recurrent/progressive IDH1-mutant (R132) glioma | Multicentre, open-label, dose-escalation, phase I study | 125-1400 mg twice daily | Treatment-related grade ≥ 3: n = 20 (42.6): neutropenia (n = 6 12.8%), diarrhoea (n = 2, 4.3%), arthralgia (n = 1, 2.1%), headache (n = 1, 2.1%), raised liver function tests (n = 5 10.7%) and hypophosphatemia (n= 2 4.3%). | ORR = 17.1% (enhancing tumors), 33.3% (non-enhancing tumors) |

Supplementary Table 5: IDH305 in clinical trials

| **Reference** | **Number of Patients** | **Disease Type** | **Phase of Trial** | **Single agent/ combined** | **Adverse effects** | **Efficacy Data** |
| --- | --- | --- | --- | --- | --- | --- |
| DiNardo, Schimmer et al (2016)  (105) | 81 | Glioma (n=32), AML (n=21), MDS (n=3), other/non-CNS solid tumors (n=24), and unknown (n=1) | Phase 1 | Single agent dose escalation | Treatment-related grade ≥ 3: elevated bilirubin 4/81 (49%),  Elevated lipase 1/81 (1.2%), rash 1/81 (1.2%) |  |
| DiNardo, Hochause et al (2023) (127) | 41 | IDH1^R132^-mutant AML/MDS | Phase I | Single agent dose escalation | Treatment-related grade ≥ 3: differentiation syndrome and tumor lysis syndrome (n = 3, 7.3% each) | CR/Cri 10/37 (27%) in AML, 1/4 (25%) in MDS |

Supplementary Table 6: Enasidenib in clinical studies

| **Reference** | **Number of Patients** | **Disease Type** | **Phase of Trial** | **Single agent/ combined** | **Toxicity** | **Efficacy Data** |
| --- | --- | --- | --- | --- | --- | --- |
| Pollyea, Tallman et al (2019)  (131) | 39 | Older adults with AML not fit for standard chemotherapy | Phase I/II | Single agent | Grade ≥3 TEAEs anaemia (5/39, 13%), indirect hyperbilirubinemia (5/39, 13%), IDH differentiation syndrome (4/39, 10%), thrombocytopenia (3/39, 8%), tumor lysis syndrome (3/39, 8%), leukopenia (2/39, 5%) and lipase increase (2/39, 5%) | ORR 12/39 (30.8%) |
| Stein, DiNardo et al (2021) (99) | 93 | mIDH1 or mIDH2 newly diagnosed AML | Phase 1, multicentre, open-label study enrolled | Enasidenib 100 mg once daily  in combination with cytarabine (200 mg/m^2^ per day for 7 days) and either daunorubicin (60 mg/m^2^ per day for 3 days) or idarubicin (12 mg/m^2^ per day for 3 days). | Grade ≥3 QT prolongation n=7 (7.5%) during induction, n = 3 (6.5%) during 3 patients (6.5%). | CR: 47% CR/CRi/CRp: 63%, at the end of the induction |
| De Botton, Montesinos et al (2023) (130) | 158 | Late-stage, mutant-IDH2 AML relapsed/ refractory to 2 or 3 prior AML-directed therapies | Open label, randomized, phase 3 trial | Single agent enasidenib or  azacitidine, cytarabine, or supportive care | Grade ≥3 TEAEs 74/158 (47%) in enasidenib arm. Increased bilirubin 13/158 (8.2%), febrile neutropenia 4/157 (2.5%), DS (14/158 8.8%) | ORR 40.5% enasidenib vs 9.9% placebo |

Supplementary Table 7: Vorasidenib in clinical trials

| **Reference** | **Number of Patients** | **Disease Type** | **Phase of Trial** | **Single agent/ combined** | **Toxicity** | **Efficacy Data** |
| --- | --- | --- | --- | --- | --- | --- |
| Mellinghoff, Penas-Prado et al (2021)  (106) | 93 | mIDH1/2 solid tumors, including 52 patients with glioma that had recurred or progressed following standard therapy | Open label  Phase 1  Dose escalation study | Vorasidenib orally, once daily | Dose-limiting toxicities of elevated transaminases occurred at doses ≥100 mg and were reversible | Non-enhancing gliomas: ORR = 18%, median PFS 36.8 months.  Enhancing gliomas: No patients had a confirmed radiographic response; median PFS 3.6 months |
| Mellinghoff, van den Bent et al (2023)  (26)  INDIGO trial | 331 (168 vorasidenib, 163 placebo) | Residual or recurrent grade 2 IDH-mutant glioma who had undergone no previous treatment other than surgery | Double-blind, phase 3 trial | Oral vorasidenib (40 mg once daily) or matched placebo | Grade ≥3 AE 22.8% vorasidenib, 13.5% placebo.  Grade ≥3 AE increased ALT in 9.6% | Median PFS 27.7 months (vorasidenib) vs. 11.1 months (placebo) |
| DiNardo, De Botton et al (2023)  (135) | 46 | mIDH1/2 advanced hematologic malignancies | open-label, first-in-human, phase 1, dose-escalation | 7 dose levels  tolerable up to doses of 600 mg QD | Study withdrawal due to AEs: AML10/34 (29.4%)  MDS 2/11 (18.2%)  4/46 (8.7%)  >/= grade 3 TRAE: diarrhoea n = 2 (4.3%), GI haemorrhage n = 4 (8.7%), increased ALT n = 4 (8.7%) | Best overall response (BOR) in AML: morphologic leukaemia-free state (MLFS) in 2/34 (5.9%). 22/34 (64.7%) SD; 6/34 (17.6%) PD; response not evaluable in 4/34 (11.8%). ORR 5.9% AML, 36.4% MDS, 27.3% for prior IDH inhibitor−naive patients |
